# An easy methodology for frost tolerance assessment in olive cultivars

**DOI:** 10.3389/fpls.2024.1397534

**Published:** 2024-07-08

**Authors:** Pedro Valverde, Enrico Maria Lodolini, Veronica Giorgi, Maria Teresa Garcia-Lopez, Davide Neri

**Affiliations:** ^1^ Department of Agricultural, Food and Environmental Sciences, Marche Polytechnic University, Ancona, Italy; ^2^ Agronomy Department, University of Cordoba, Escuela Tecnica Superior de Ingenieros Agronomos y de Montes (ETSIAM), Cordoba, Spain; ^3^ Research Centre for Olive, Fruit and Citrus Crops, Council for Agricultural Research and Economics, Rome, Italy

**Keywords:** acclimation, cold, detached leaves, frost damages, olive cultivars, *Olea europaea*

## Abstract

**Introduction:**

Olive cultivation, like other evergreen fruit crops worldwide, is limited by the occurrence of frost episodes in different times of the year, mainly in winter or early spring. Some contradictory results are reported about cultivars’ response to frost, which depends on the physiological stage of the tissues (acclimated or not acclimated) when the cold or frost episode occurs. This work aimed to implement a user-friendly and reliable lab method for discerning frost tolerance.

**Methods:**

Our methodology considered both detached leaves and potted plantlets. The optimal temperature at which damage differentiated between cultivars was evaluated, as well as the time of exposure to cold and the recovery time for the correct evaluation of the symptoms. Furthermore, a comparative analysis of damage on both young and mature leaves was conducted. To validate the efficacy of the methodology, assessments were conducted on the cultivars ‘Arbequina’ (tolerant), ‘Picual’ (moderately tolerant), and ‘Frantoio’ (susceptible) under acclimated and non-acclimated conditions.

**Results and discussion:**

The results indicated that, when detached leaves were used for frost evaluation, a temperature of -10°C ± 1°C for 30 min and a recovery time at 26°C for 24–48 h after exposure to cold are enough to induce damages on the leaves and discriminate between cultivar susceptibility. Under these conditions, a precise assessment of symptoms can be made, facilitating the categorization of frost tolerance level in various olive cultivars. Notably, no significant differences were observed between young and mature leaves during the evaluation process. On the other hand, the critical temperature to assess damages on potted plantlets was determined to be -7°C ± 1°C. In addition, it was observed that acclimated plants exhibited fewer symptoms compared to non-acclimated ones, with ‘Frantoio’ being the most affected alongside ‘Picual’ and ‘Arbequina’.

**Conclusion:**

The implemented methodology will allow the assessment of frost tolerance in several olive cultivars within a short timeframe, and it is proven to be user-friendly and reliable.

## 1 Introduction

Olive is the most widely cultivated fruit crop worldwide, spanning 10.3 million ha (Food and Agriculture Organization FAOSTAT, 2021) predominantly located between the 30° and 45° parallels in the northern and southern hemispheres. Olive cultivation thrives in regions where the climatic conditions are similar to those of the Mediterranean Basin. Over millennia, farmers have selected and propagated cultivars based on their adaptability to specific geographical and pedo-climatic conditions (Civantos, 2008; Diez et al., 2015; Lucena et al., 2017; Rallo et al., 2018). Within the favorable latitudes for olive cultivation, the Mediterranean Basin claims approximately 70% of the global olive-cultivated area. This dominance can be attributed to the climate, characterized by mild, rainy winters and dry, hot summers representing the best conditions for ensuring regular fruit production. It is worth noting that the Mediterranean Basin is not only the area where the olive tree was domesticated but also the one hosting the largest populations of wild olive trees.

Within these favorable areas for the cultivation of this crop, several biotic (pests and diseases) and abiotic stresses (edaphoclimatic agents) can affect the olive tree (Barranco, 2017). In this sense, one of the abiotic factors strongly affecting the development of the olive crop is tolerance to low temperatures (frost events), which determines the viability of the crop (Bartolozzi and Fontanazza, 1999; Petruccelli et al., 2022).

Frost tolerance has been studied both in annual crops (Cattivelli and Crosatti, 2020) and in woody species (Warrington and Rook, 1980), and it is commonly influenced by the physiological state of the plant, with much less damage being observed in acclimated plants. In both annual and perennial crops, field evaluations in different locations are reported to be the most reliable system to assess frost tolerance, although the development of evaluation methods under controlled conditions may allow faster tests to categorize frost tolerance in different species and varieties (Warrington and Rook, 1980; Cattivelli and Crosatti, 2020). In annual crops such as cereals (wheat, barley, or rye), one of the most commonly used evaluation methods consists of the field assessment of the damage caused by low temperatures using a visual damage scale, normally comprised between 0 and 9. In these crops, the phenological stage at the time of frost exposure is of great importance since the damage results are of a different entity, with more serious damages normally observed when the plant is flowering (Cattivelli and Crosatti, 2020). In the case of woody species, various authors evaluated frost tolerance both in controlled and field conditions (Warrington and Rook, 1980). Frost tolerance in olive has been studied for decades, particularly focusing on understanding the response of the different cultivars to frost events. This emphasis is due to the large number of olive orchards planted in border areas, where climatic conditions are conducive to such occurrences. Additionally, the response to cold episodes contributes to understand the crop adaptation under varying climatic conditions (Palliotti and Bongi, 1996; Bartolozzi and Fontanazza, 1999; Barranco and Gómez-del Campo, 2005; Lodolini et al., 2016; Valverde et al., 2020). Furthermore, olive is undergoing an expansion outside the traditional cultivation areas, and new cultivars have been developed through breeding programs whose adaptation or tolerance to low temperatures is unknown (Rallo et al., 2008; Camposeo et al., 2021; Valverde et al., 2023). The frost tolerance of olive trees hinges on the intensity and timing of the freezing events. In fact, the olive tree is an evergreen species that undergoes an acclimation process to face low temperatures. However, if the freezing event is intense and occurs before the completion of the acclimation process, it can pose significant risks to the different tissues of the tree (Bartolozzi et al., 2002; Cansev et al., 2009).

Acclimation in the olive tree involves a series of physical and biochemical changes regarding the restructuring of cell membranes, the accumulation of cryoprotectants, and the alterations in F-actin (D’Angeli et al., 2012). For acclimation to occur, it requires the accrual of cold, a process that requires the gradual reduction of temperature as observed during the autumn season (Guerra et al., 2015). However, this process does not imply a total tolerance of the olive tree when temperatures fall below 0°. Several studies assessed the frost temperature inducing damages in olive trees, albeit with variations in identified threshold temperatures. Palliotti and Bongi (1996) determined that the temperatures below -7°C adversely affected the aerial part of the plant and observed damages in the entire tree, which could lead to death, with temperatures below -12°C (Larcher, 1970). In parallel, Barranco and Gómez-del Campo (2005) settled the threshold at -8.6°C, reporting that an olive tree can suffer acute damage at this temperature. Other studies examined cold events occurring after de-acclimation, during vegetative restart, considering spring frosts that follow unusual warm periods in late winter. In such scenario, damages are significantly more pronounced—for instance, temperatures ranging from -4.7°C to -6.4°C (Lodolini et al., 2016) and -7.6°C (Valverde et al., 2020) resulted in heavier damages when compared to those observed when these temperatures occurred after full acclimation the tree.

Plant tissues also exhibit different responses to freezing events. Winter cold tended to impact leaves, shoots, and branches more intensely, while spring frosts could injure inflorescences or fruits, exerting a more direct influence on the quantity and quality of crop production (Pino et al., 2022). Zucchini et al. (2023a) reported visible symptoms on the inflorescences after a late spring frost event (early April) occurred in central Italy, with losses of up to 95% in some of the studied cultivars and indicating 2°C as the dangerous threshold for the inflorescences in this phenological stage. Furthermore, the frost damages caused in different tissues are directly related to the extension of damages caused by other diseases, such as olive knot caused by *Pseudomonas savastanoi* pv. Savastanoi (Valverde et al., 2020; Zucchini et al., 2023b).

For decades, the assessment of the cold tolerance of the cultivars has been studied in olive (Bartolozzi and Fontanazza, 1999; Barranco and Gómez-del Campo, 2005; Lodolini et al., 2016, 2022), even though contradictory results have been reported. This is due to a lack of knowledge of the specific requirements for each cultivar to achieve full acclimation and de-acclimation. Moreover, there are a series of factors determining the level of tolerance of a plant to cold conditions. The significance of frost tolerance in olive trees is widely acknowledged, with differences between cultivars playing a crucial role in determining this trait—that is, depending on the cultivar, the physiological and phenotypic response to these episodes is different (Barranco and Gómez-del Campo, 2005; Lodolini et al., 2016; Valverde et al., 2020; Mete et al., 2023). In general, olive trees with an unbalanced nutritional status suffer more damages caused by different biotic or abiotic stresses (Fernández-Escobar, 2019). Saadati et al. (2021) determined that the application of potassium a few months before the onset of winter can trigger a sequence of biochemical reactions, enhancing the cold tolerance of the ‘Rashid’ cultivar. Conversely, Fernández-Escobar et al. (2011) observed a correlation between nitrogen (N) concentration in olive leaves and cold tolerance. In October (northern hemisphere), leaves with higher N content showed increased stress tolerance, while during spring vegetative restart, N-deficient trees were more tolerant. On the contrary, no significant differences were found in winter based on leaf N content. Furthermore, D’Angeli et al. (2003) determined that calcium concentration might play a role in the adaptation of the olive tree to cold episodes.

Another factor directly related to the response of the tree to frost episodes is the level of hydration of the different tissues that can be determined, in part, by the type of soil and by agronomic management. The on/off crop load year, which largely determines the nutritional state of the tree, can affect the response of the tree to frost. Turchetti-Iturrieta et al. (2014) observed that the water status of the tree in the ‘Arbequina’ and ‘Barnea’ cultivars can modify the frost tolerance and that the trees subjected to water stress better tolerated low temperatures.

Some studies focused on evaluating the tolerance to frost of the cultivars in field conditions and at the laboratory level. In field assessments, the damages observed in olive trees after an episode of temperatures below 0°C are normally evaluated. Barranco and Gómez-del Campo (2005) carried out the damage evaluation after a frost episode in the field by weighing the affected and unaffected part of the plant. They classified the resistance of eight olive cultivars as percentage of affected tissue, concluding that ‘Cornicabra’, ‘Arbequina’, and ‘Picual’ were the most tolerant varieties, while ‘Empeltre’ was the most susceptible one. On the other hand, Lodolini et al. (2016), when evaluating 24 olive cultivars through the visual index of trees’ defoliation and bark split, concluded that ‘Ascolana Dura’ and ‘Orbetana’ had the best performance, while ‘Arbequina’, ‘Fs-17’, ‘Raggia’, and ‘Sargano di San Benedetto’ were the most affected.

In the laboratory, the most commonly used technique to evaluate frost damage is “electrolyte leakage (EL)”, introduced by Dexter et al. (1930), which hinges on the principle that cells sustaining damage lose their ability to regulate their membranes effectively, leading to the release of electrolytes through these compromised membranes (Aslani-Aslamarz et al., 2011). EL yields sigmoidal curves for each leaf sample (consisting of small discs), illustrating the impact of consecutive subzero temperatures on releasing cell electrolytes (CEr, relative electrical conductivity) into an aqueous medium. The temperature at which the curve inflection point occurs (LT50) is the metric used to compare among cultivars. EL analysis has been used quite effectively to evaluate frost damage in several species like grape canes and buds, red raspberry canes, blueberry shoots, etc., although it is recommended to compare the results obtained through various methodologies to ensure a comprehensive and accurate tolerance evaluation (Yu and Lee, 2020).

In the case of the olive tree, some researchers used the EL technique to classify cultivars attending to their frost tolerance (Bartolozzi and Fontanazza, 1999; Barranco and Gómez-del Campo, 2005; Azzarello et al., 2009; Saadati et al., 2019; Rezaei and Rohani, 2023). Bartolozzi et al (1999). evaluated 13 cultivars and classified ‘Bouteillan’ and ‘Nostrale di Rigali’ as the most tolerant to frost, while ‘Ascolana tenera’, ‘Frantoio’, and ‘Leccino’ resulted in similar values of LT50 (-15.1°C, -14.3°C, and -14.3°C, respectively). With the same methodology, Barranco et al (2005). evaluated eight cultivars and determined ‘Cornicabra’, ‘Picual’, and ‘Arbequina’ as the most tolerant cultivars, while no significant differences were found when comparing ‘Arbequina’ with ‘Frantoio’; the latter was known for its susceptibility to cold and frequently used as a susceptible “control” in the experimental designs. In this sense, in the field experiment carried out to compare the percentage of affected tissue, significant differences were obtained between the ‘Arbequina’ and ‘Frantoio’ cultivars (Barranco and Gómez-del Campo, 2005). Bartolozzi and Fontanazza (1999) and Azzarello et al. (2009) evaluated the frost tolerance of 21 cultivars, using both non-acclimated and acclimated plants, and determined ‘Coratina’, ‘Campleglio’, and ‘Zamarian San Rocco’ as the least tolerant cultivars, while ‘Frantoio’, ‘Leccino’, and ‘Ascolana tenera’ showed no differences. This method requires time and a specialized lab and cannot be done simultaneously for a high number of genotypes.

The findings described in the previous paragraphs show some incoherent behaviors between cultivars, highlighting the importance of comparing cultivars under homogeneous conditions and considering other factors such as nutritional status, size, and age of the plant and water status since variations in these parameters will directly influence the results. The studies carried out so far showed differences between field and controlled-condition assessments and the difficulty of evaluating several cultivars in the same conditions. In fact, in a field experiment, environmental conditions are difficult or impossible to control, and the variability between sites and plants is high. Potted plants are more easily kept at similar conditions, but an easy and fast method is required if a high number of genotypes needs to be evaluated. That is especially important in olive breeding programs for the early selection of new cultivars with interesting agronomic characteristics. The need for the implementation of a method for an easy and quick evaluation of a large number of olive cultivars and genotypes is highly required.

In this sense, our experiments focused on (i) defining the optimal temperature at which symptoms appear in olive leaves, ii) comparing what kind of olive leaves are the most suitable for the evaluation. As a result, the present study introduces a novel methodology for assessing frost tolerance in olive tree under controlled conditions.

## 2 Materials and methods

For the development of the methodology for evaluating frost tolerance in olive plants, two experimental layouts were designed under laboratory conditions: the first one consisted in the assessment of detached leaves and the second one in the assessment of potted plantlets.

### 2.1 Detached leaves

Plants from six olive cultivars (‘Arbequina’, ‘Arbosana’, ‘Ascolana Tenera’, ‘Frantoio’, ‘Leccino’, and ‘Picual’) were grown in a greenhouse with homogeneous parameters (28°C ± 2°C, 60%–70% relative humidity, 12-h photoperiod with 400 μmol·m^-2^·s^-1^) and were used depending on their frost tolerance according to different bibliographical sources (Bartolozzi and Fontanazza, 1999; Barranco and Gómez-del Campo, 2005; Lodolini et al., 2016; Barranco, 2017).

Two leaves were detached from each of the 10 olive plantlets per cultivar (6 months old, 60-cm height, and potted in 7 × 7 × 8 cm from a semi-hardwood cutting). Sampling was performed in July on the central portion of plantlets. The plantlets were cared for homogenously to avoid differences in water stress and nutritional status. The detached leaves were fixed on a plastic sheet (A4 format) using a metal clip and showing both the beam and the underside (10 leaves per face) to evaluate the symptoms of frost on the upper (adaxial) and lower (abaxial) surfaces of the leaf. The exposure to frost consisted of the introduction of the detached leaves fixed on plastic sheets in a horizontal freezer cabinet at specific temperatures and times. Each plastic sheet (containing 20 leaves) was considered as a block, and a three-block design was considered in each trial.

In a first experiment, frost treatments were performed by keeping the same temperature (-18°C ± 1°C) and testing different exposure times (15, 30, 45, 60, 120, and 180 min compared with the control, 0 min of exposure); the leaves were positioned in the freezer when the thermometer reached the target temperature. In a second experiment, the same exposure time was maintained (30 min), while different temperatures below 0°C (i.e., -6°C ± 1°C, -8°C ± 1°C, -10°C ± 1°C, and -18°C ± 1°C) were assayed. Leaves that were not exposed to frost were always included as a control, using the same number of leaves as that used in the frost treatments, and pinned to plastic sheets as the other treatments but kept at room temperature.

**Figure 1:**
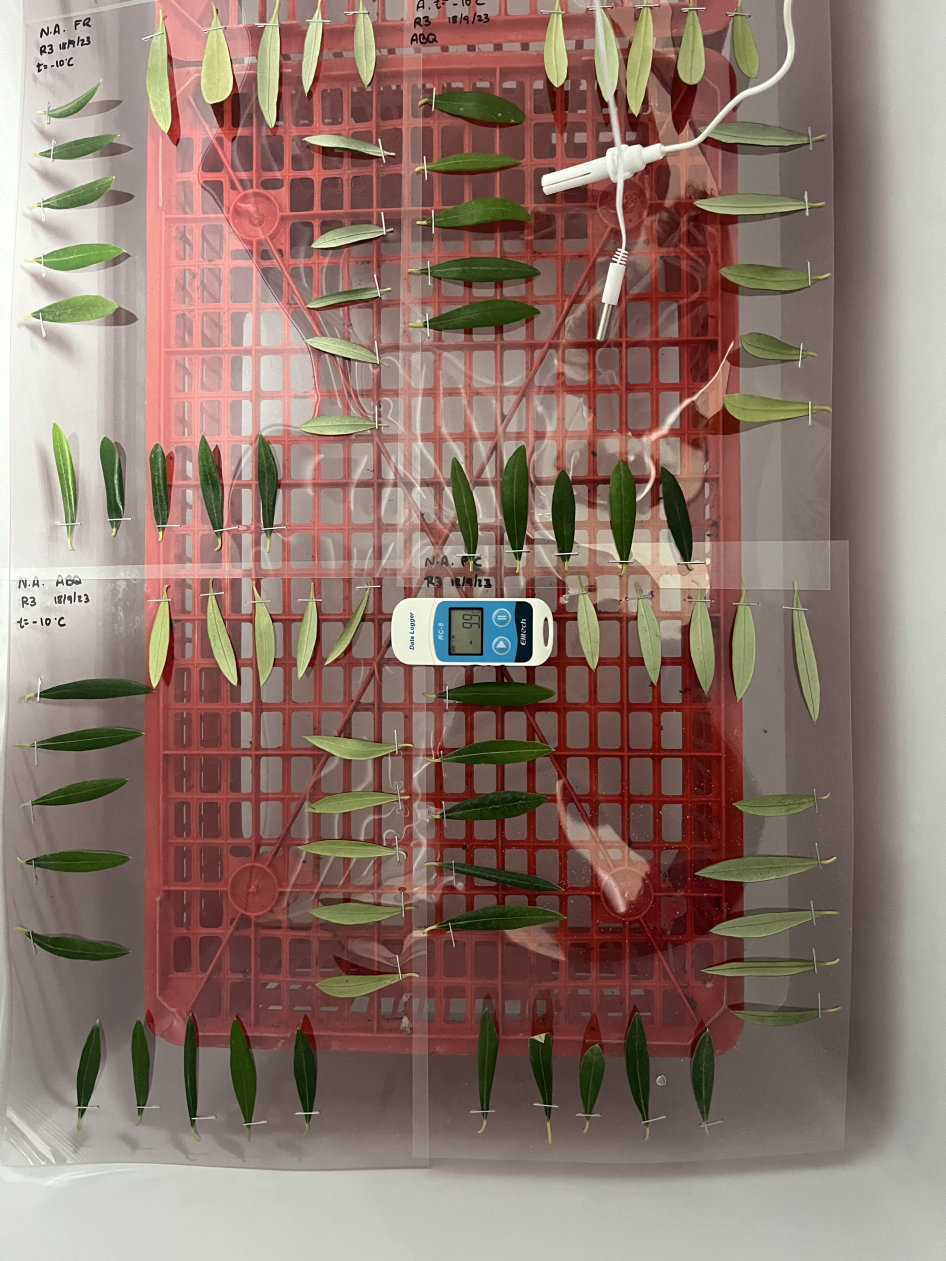
Disposition of the detached leaves, fixed on a plastic sheet (A4 format) using a metal clip and showing both the adaxial and abaxial surfaces (10 leaves per side, for a total of 20 leaves per cultivar), when exposed to frost treatments in a horizontal freezer cabinet. The plastic sheets were placed at the same height to avoid temperature gradients. A datalogger and temperature sensors were placed at the same level to monitor the temperature.

**Figure 2:**
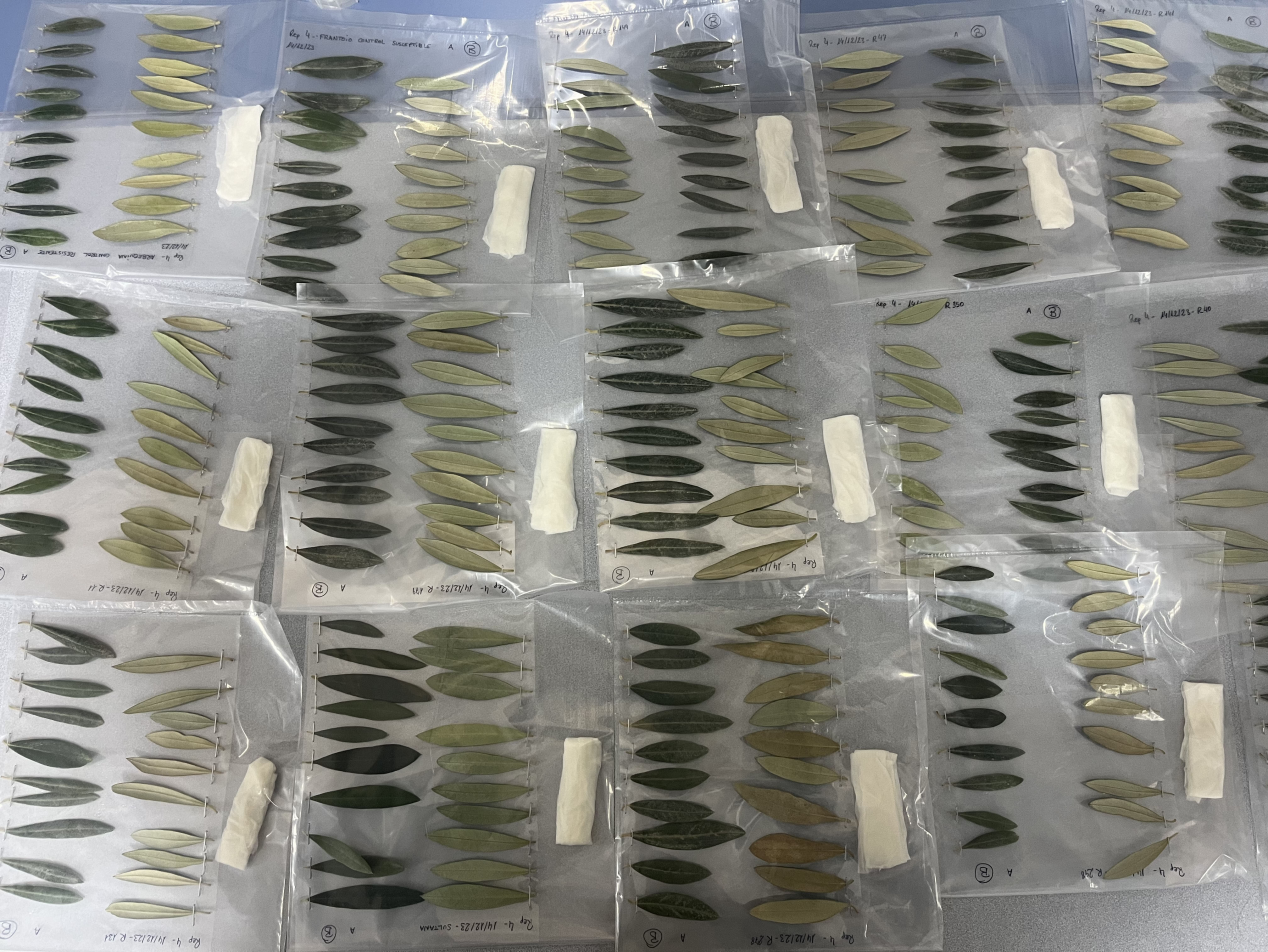
Leaves of different cultivars in 26°C and high-humidity chambers (100% relative humidity), awaiting the onset of frost symptoms.

The temperature was monitored continuously with thermometers whose sensors were placed at the same height as the leaves and connected to a datalogger (**Figure 1**). To ensure consistent exposure to the cold within the freezer, the plastic sheets with the leaves were positioned horizontally at the same height, thus avoiding temperature gradients inside the freezer. After exposure to frost, the sheets with the leaves were removed from the freezer and, for the correct manifestation of the symptoms, were placed in high-humidity chambers with a temperature of 26°C ± 2°C. Control leaves were also put in the humidity chambers at the same time as the other treatments. The humidity chambers consisted of closed plastic bags moistened with paper to achieve 100% relative humidity, avoiding the natural drying of the detached leaves and correctly observing the damage caused by the freezing of the tissues (**Figure 2**).

**Figure 3:**
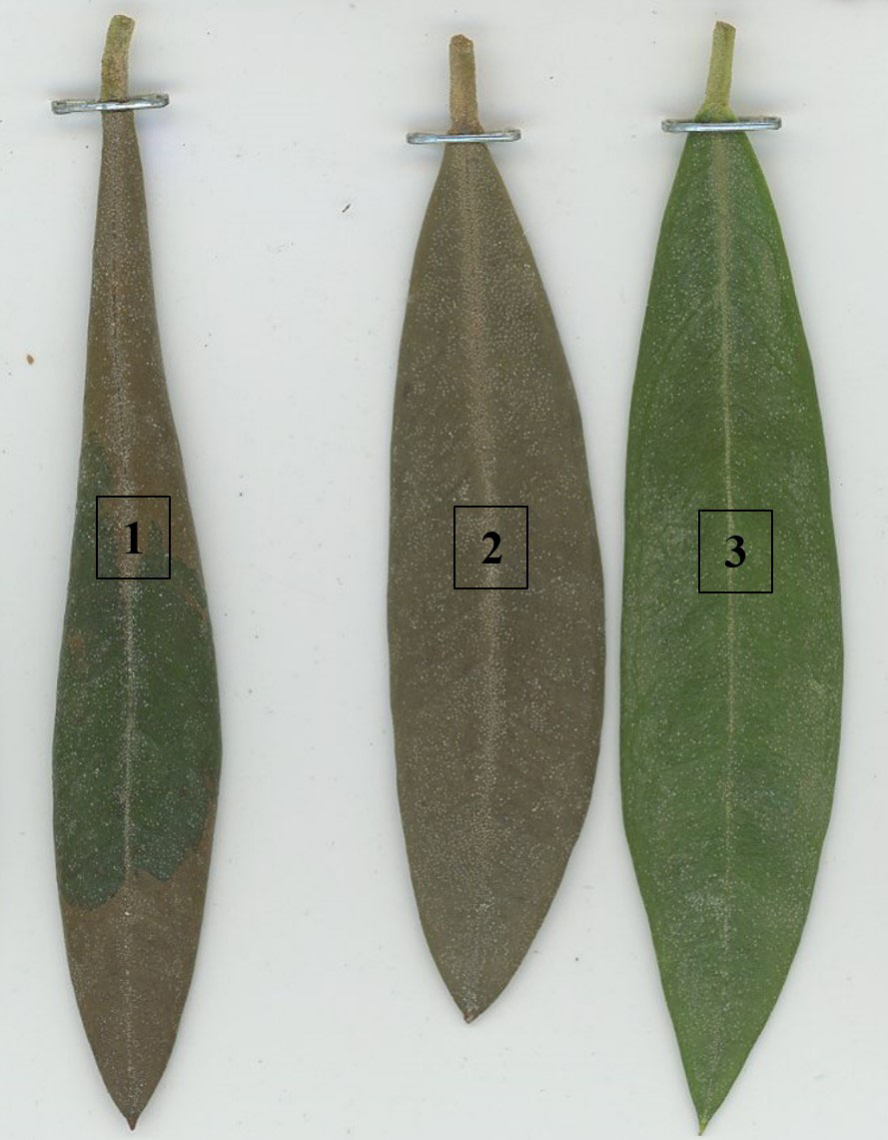
Observed symptoms in three leaves 96 h after frost exposition at -10°C for 30 min in ‘Arbequina’ cultivar: (1) affected, (2) dead, and (3) healthy.

Symptom evaluation was conducted over the adaxial surface (leaf beam) and abaxial surface (leaf underside) of the leaves 24, 48, 72, 96, and 172 h after the frost treatments. The number of affected dead and healthy leaves, respectively, was recorded to calculate the incidence (percentage of damaged leaves = affected + dead) and mortality (percentage of dead leaves) (**Figure 3**). To determine whether the leaf was healthy, affected, or dead, the entire leaf was observed, including the central vein.

At the same time, a comparison between the performance of fully expanded leaves, i.e., mature (from the middle portion of the plantlets), and newly formed ones (young leaves from the third to the fourth apical nodes) was conducted, exposing them to the same conditions of the second approach, while symptom evaluation was assessed 48 h after frost exposition.

### 2.2 Potted plantlets

The potted plant evaluation consisted of two experiments. Firstly, to check the correct temperature to evaluate the symptomatology, the cultivars ‘Arbosana’ and ‘Leccino’ were exposed to -18°C ± 1°C, -10°C ± 1°C, -7°C ± 1°C, and -5°C ± 1°C. A second test consisted of the exposure temperatures of -6°C ± 1°C, -7°C ± 1°C, and -8°C ± 1°C using the cultivars ‘Arbequina’, ‘Arbosana’, ‘Ascolana tenera’, ‘Frantoio’, ‘Leccino’, and ‘Picual’. Each experiment was conducted in triplicate, with two plants assessed for each cultivar in every trial. For both experiments, the exposure time to cold in the freezer was 30 min, and the evaluation of symptoms (frost damage, incidence, and mortality) was carried out 48 h after exposure.

**Figure 4:**
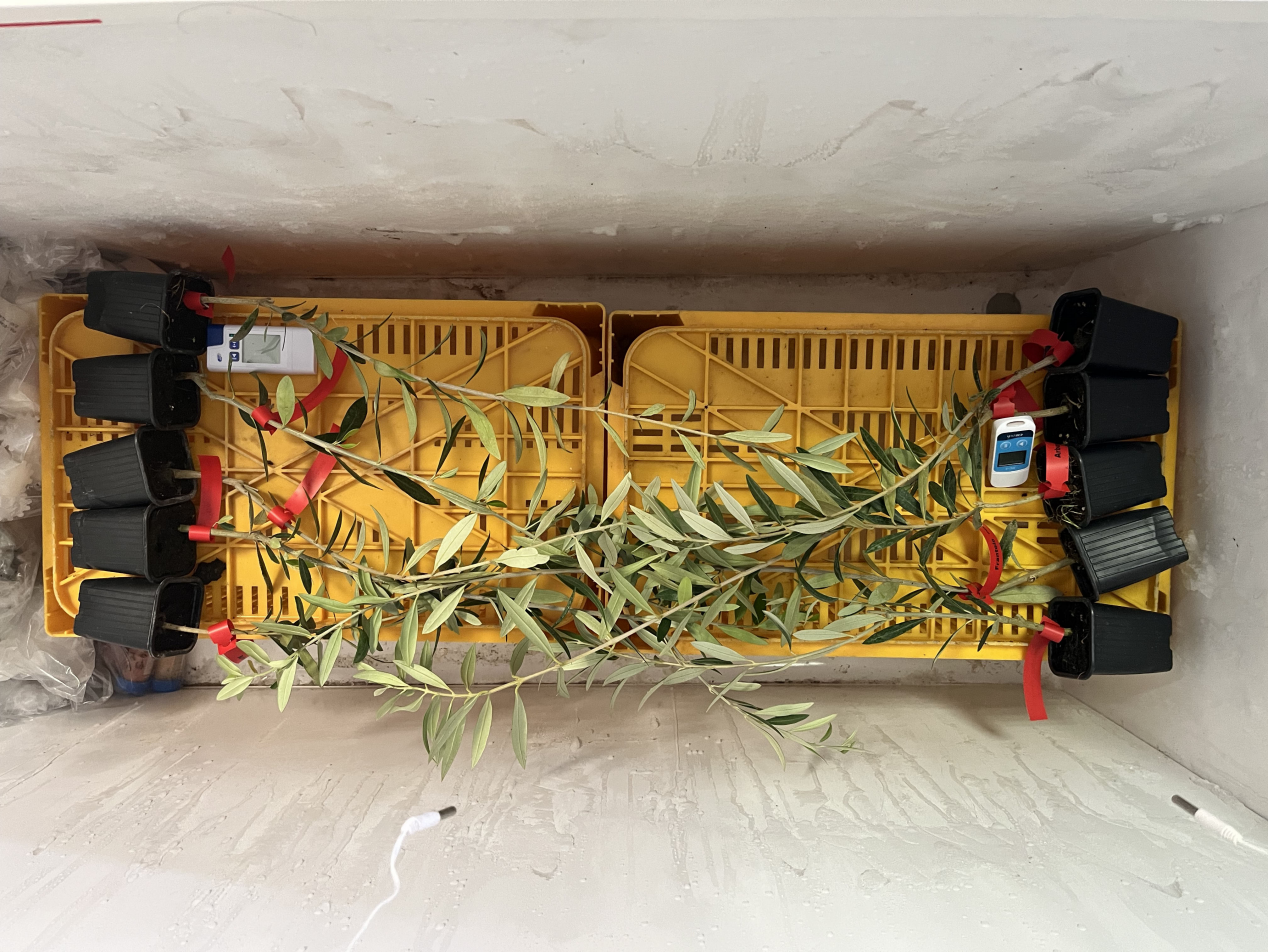
Trial of exposition to frost using potted olive plantlets. Trial of exposition to frost using potted olive plantlets placed horizontally at the same height in the freezer to avoid temperature gradients and using datalogger and temperature sensors to monitor the temperature.

The same horizontal freezer cabinet, as detailed above, was used to expose the potted plantlets to frost treatments. In this case, the plantlets were placed horizontally, at the same height, to guarantee the same temperature distribution among the tissues, thus avoiding temperature gradients inside the freezer. The temperature was monitored with thermometers whose sensors were placed at the same height as the plantlets and connected to a datalogger (**Figure 4**).

The exposure time to frost began when the thermometer reached the target temperature. After exposure to frost, the plantlets were removed from the freezer and placed in high-humidity chambers with a temperature of 26°C ± 2°C for the correct manifestation of the symptoms. The symptom evaluation consisted of observing the number of dry leaves (leaves with brown coloration and curling) on each plant 48 h after the frost exposition. The parameters considered were incidence (percentage of plants with dry leaves) and mortality (percentage of plants with 100% of dry leaves).

### 2.3 Acclimated and non-acclimated plantlets

After establishing the frost tolerance evaluation methodology, its reliability was verified by employing three olive cultivars with different frost tolerance: ‘Arbequina’ and ‘Picual’, classified as high-intermediate tolerant, and ‘Frantoio’, classified as low-tolerant cultivar, based on the studies of Bartolozzi and Fontanazza (1999) and Barranco and Gómez-del Campo (2005). The experiment aimed to compare the response of the cultivars to frost treatments in both acclimation and no-acclimation conditions. To achieve this, 12 potted plantlets per cultivar underwent a 6-week acclimation period in a photoperiod-controlled room (12-h photoperiod, 400 µmol·m^-2^·s^-1^) at 8°C–10°C. Concurrently, the same number of plantlets per cultivar was maintained in a separate growth chamber with similar daylight and relative humidity conditions (12 h and 60%–70%, respectively) but at 28°C ± 2°C. The plantlets used in this experiment had the same characteristics as those used in the previous ones.

In this case, 20 detached leaves per cultivar were simultaneously evaluated, sourced from both acclimated and non-acclimated plantlets. The leaves were randomly detached from the 12 plantlets of each cultivar. The test was replicated three times, cataloging each repetition as a block for subsequent statistical analysis. The parameters selected to perform the frost treatment were those optimized before, and the freezing temperature was at -10°C ± 1°C with the detached leaves set in plastic sheets and placed inside the horizontal freezer cabinet for 30 min. Frost damages were evaluated 48 h after frost exposition using the methodology described before.

### 2.4 Statistical analysis

The numbers of affected and dead leaves for the evaluated parameters (exposure time to frost, temperature, leaf adaxial and abaxial surfaces, adult and young leaves, time of evaluation after frost exposure, and acclimated and non-acclimated leaves), respectively, were subjected to the analysis of variance and subsequently to Tukey’s HSD means comparison test with a significance level of 0.05. The percentages of incidence and mortality in the potted plantlet trial were analyzed using chi-square test with a probability level of 0.05. All of the analyses were developed using the statistical program Statistix 10 (Analytical Software, 2105 Miller Landing Rd., Tallahassee, FL 32312, USA).

## 3 Results

### 3.1 Detached leaves

**Figure 5:**
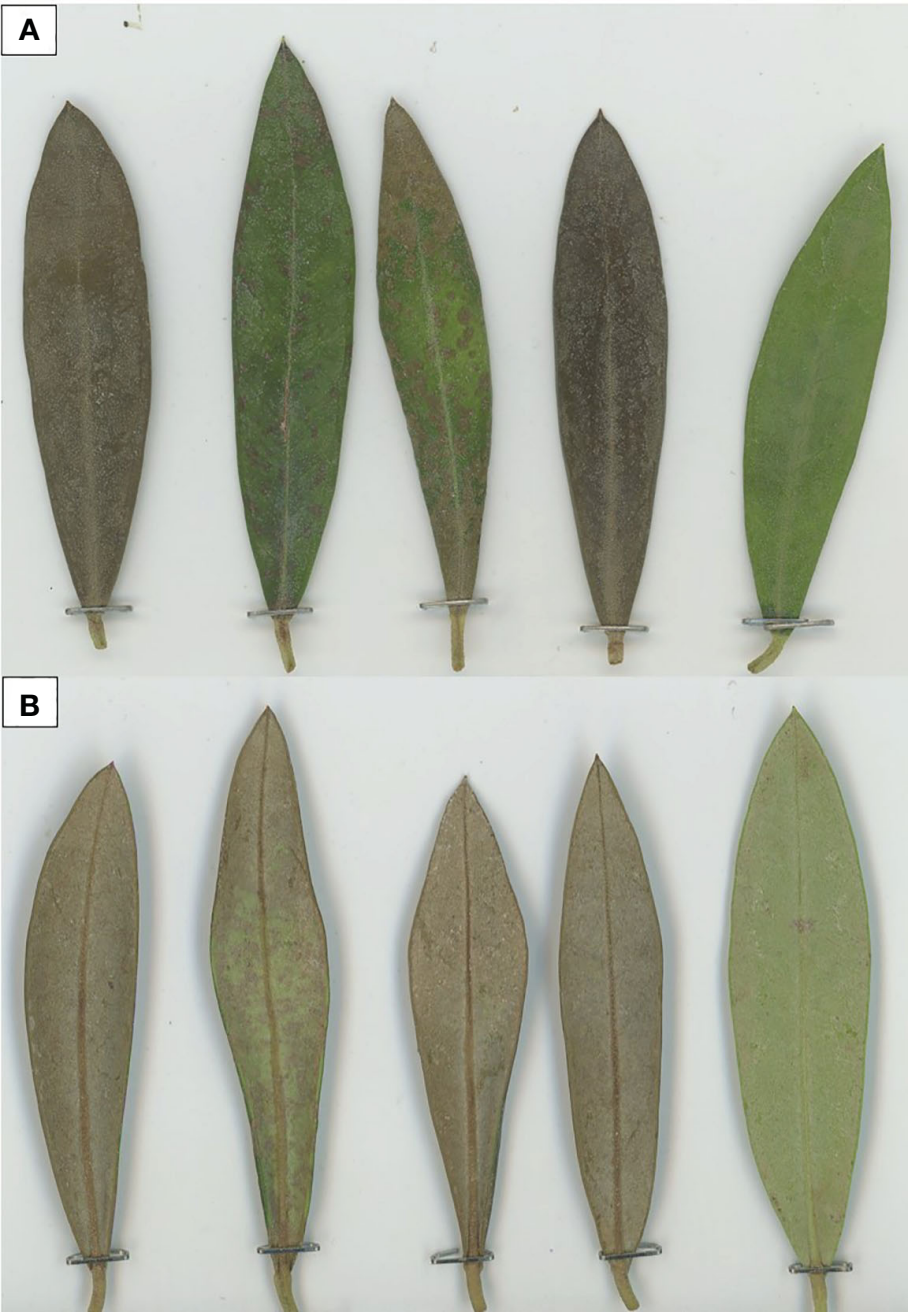
Frost damage symptoms observed in the adaxial (upper, **A**) and abaxial (lower, **B**) surfaces of the leaves. Note the total or partial coloration of the upper side of the leaf **(A)** and the brown discoloration of the nerve of the lower side of the leaf **(B)**.

In general, the observed symptoms on the leaves appeared to be like those observed in frost episodes in the field, including a change of color from intense green to deep brown, usually associated with desiccation of the leaves. In our tests, green color, partial browning of the leaf surface (partial death of the leaf cells), and brown color indicated not damaged, partially damaged, and dead leaves, respectively (Lodolini et al., 2016; Petruccelli et al., 2022) (**Figure 5**).

All of the leaves subjected to -18°C ± 1°C were dead when exposed for 30, 45, 60, 120, and 180 min in all the cultivars evaluated, while no dead leaves were found after 15 min. This result indicates that 30 min of frost exposure is enough to elicit visible symptoms in the leaves. Consequently, this specific duration was selected for the subsequent experiments (**Table 1**).

**Table 1:** Average incidence (%) and mortality (%) obtained 48 h after frost exposition when testing different exposure times, maintaining -18°C, and when testing different temperatures, with a consistent exposure time of 30 min, on detached leaves.

Trial	Time	Subzero temperature	Incidence^a^	Mortality^a^
	(min)	(°C)	(%)	(%)
Exposure time	15	-18	0a	0a
	30	-18	100b	100b
	45	-18	100b	100b
	60	-18	100b	100b
	120	-18	100b	100b
	180	-18	100b	100b
Temperature	30	-6	0a	0a
	30	-8	0a	0a
	30	-10	80b	70b

*Different letters indicate significant differences between the data with a *P* = 0.05, according to Tukey’s HSD Test.

To determine the critical temperature to induce symptoms in the leaves, the same exposure (30 min) and different temperature levels (-6°C ± 1°C, -8°C ± 1°C, and -10°C ± 1°C) were compared. The temperatures of -6°C and -8°C did not induce symptoms on the detached leaves in all tested cultivars and for both the adaxial and the abaxial leaf surfaces. Such a result was observed even 1 week after the frost exposure (preserving the detached leaves in high humidity and 26°C ± 1°C). On the contrary, at -10°C ± 1°C, a different symptom manifestation was observed in the leaves between the tested cultivars (**Table 1**). Complete or partial browning was detected across the entire adaxial surface of the leaves. However, on the abaxial surface, the change of color was less discernible in partially affected leaves, except when the midrib was considered, where the change was notably pronounced. This result indicates that both sides of the leaf can be suitable for the evaluation of frost symptoms, as no significant differences were found in the transition from green to brown in the adaxial and abaxial (including the midrib) surface of the leaves (**Table 2**, **Figure 5**).

**Table 2:** Average incidence (%) and mortality (%) depending on the time to symptom evaluation after frost exposition at -10°C (24, 48, 72, 96 and 192 hours) and incidence and mortality obtained comparing the leaf side (adaxial and abaxial surface) and age (adult and young leaves) after 48 hours after frost exposition.

Trial		Incidence*	Mortality*
		(%)	(%)
Leaf side	Adaxial	87.8a	74.4a
	Abaxial	92.2a	91.1a
Leaf age	Adult	81.3a	77.5a
	Young	62.5a	62.5a
Hours after frost exposure	192	93a	85a
	96	92a	85a
	72	91a	85a
	48	91a	85a
	24	88a	84a
	0	0b	0b

* Different letters indicate significant differences between the data with a P = 0.05, according to Tukey’s HSD Test.

Regarding the timing of symptom evaluation following frost exposure, no significant differences were observed in leaves between 24- and 172-h intervals. This implies that a window of 24–48 h post-frost exposure is enough for the manifestation of symptoms in the leaves (**Table 2**). Additionally, when comparing fully expanded (mature) leaves with newly formed ones (young) to evaluate the symptoms, no significant differences were found, indicating that both kinds of leaves are equally applicable to detect cultivar susceptibility to frost (**Table 2**).

Differences between the tested cultivars were found in response to the frost treatments (**Table 3**), with ‘Leccino’ as the most sensible and ‘Picual’ as the most resistant.

**Table 3:** Average incidence (%) and mortality (%) 24 h after frost exposition at -10°C for 30 min of the detached leaves of six cultivars.

Cultivar	Incidence	Mortality
	(%)	(%)
Leccino	100.0a	100.0a
Ascolana Tenera	93.6ab	69.3ab
Frantoio	91.7abc	68.3ab
Arbosana	73.3bcd	63.3ab
Arbequina	70.0cd	39.3b
Picual	61.9d	31.7b

Different letters indicate significant differences between the data with a P = 0.05 according to Tukey’s HSD test.

### 3.2 Potted plantlets

Regarding potted plantlets exposed to frost, no damage was recorded within the temperature ranges of -°C and -6°C. However, at -7°C, 36.1% of the plantlets across all cultivars were affected, leading to a mortality of 11.1%. These symptoms appeared as leaf desiccation while the shoots remained unaffected. Notably, the symptoms on the leaves attached to the plantlet were the same as those observed on detached leaves, with discoloration observed from green to brown. No significant differences were observed when the comparison between cultivars was performed (**Table 4**). When the temperature exposure was set at -8°C, 70% mortality and incidence were observed across all cultivars. Furthermore, when the temperature was established at -10°C or below (-18°C), 100% mortality was recorded across all tested cultivars.

**Table 4:** Frost damage incidence (% of affected plants) and mortality (% of dead plants) evaluated 48 h after exposition to -7°C during 30 min in the potted plantlets.

Cultivar	Plants	Incidence	Mortality
	(no.)	(%)	(%)
Leccino	6	2.0	2.0
Arbosana	6	2.0	1.0
Picual	6	2.0	0.0
Frantoio	6	3.0	1.0
Arbequina	6	2.0	0.0
Ascolana Tenera	6	2.0	0.0
Total	36	36.1	11.1

No differences in the values of incidence and mortality when applying the chi-square test (P = 0.05).

### 3.3 Acclimated and non-acclimated plantlets

**Figure 6:**
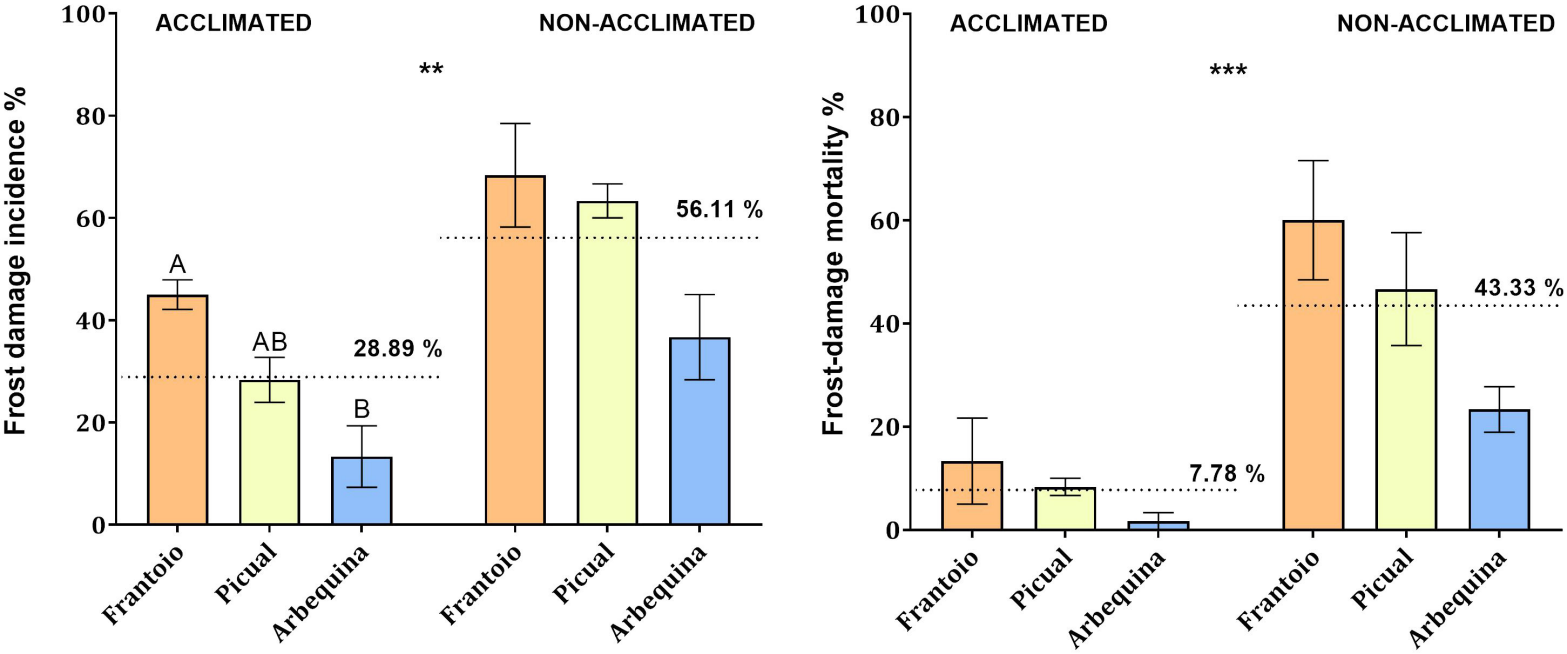
Frost damage incidence (percentage of affected leaves, left) and frost damage mortality (percentage of dead leaves, right) observed in the cultivars Frantoio, Picual, and Arbequina when detached leaves of acclimated and non-acclimated plantlets were exposed to -10°C for 30 min. Bars represent mean values and standard errors. A completely randomized ANOVA and the Tukey test allowed comparisons of the data with *P* = 0.05. ** indicate significant differences between acclimated and non-acclimated average incidence. *** indicate significant differences between acclimated and non-acclimated average mortality.

The results indicated significant differences in the response (incidence and mortality) to frost exposure of acclimated plantlets compared to non-acclimated ones in the three evaluated cultivars. Frost damage incidence was lower (*P* = 0.004) in acclimated plantlets (28.9%) than in non-acclimated ones (56.1%). Similarly, mortality was lower (*P* < 0.001) in acclimated plantlets (7.8%) compared to non-acclimated ones (43.3%). Moreover, significant results were found among acclimated cultivars when comparing their frost damage incidence (*P* = 0.008). The cultivar ‘Arbequina’ was the least affected (13.3%), and ‘Frantoio’ (45.0%) resulted as the most susceptible, while ‘Picual’ (28.3%) showed intermediate values without significant differences compared to the other tested cultivars (**Figure 6**).

## 4 Discussion and conclusions

In the present work, an easy and quick methodology to assess olive cultivars according to their level of frost tolerance was developed. The observed symptoms, both in detached leaves and potted plantlets experiments, corresponded to those observed in previous studies, both under controlled and field conditions (Karamatlou et al., 2023). Our results indicated that symptoms can be evaluated both by observing the adaxial and abaxial surface (including the midrib) of olive leaves. Concerning freezing temperature, previous studies showed that the first symptoms of damage in the leaves can be detected at temperatures below -4°C (Lodolini et al., 2016).

However, comparing the results becomes challenging due to the numerous variables influencing the olive tree response to temperatures below 0°C (Barranco and Gómez-del Campo, 2005; Fernández-Escobar et al., 2011; D’Angeli et al., 2012; Lodolini et al., 2016; Valverde et al., 2020). Throughout this work, we found that -10°C was the most appropriate temperature to subject the detached leaves in order to give rise to different symptoms among cultivars. This temperature was also determined in previous studies as a prompt for symptom appearance (Karamatlou et al., 2023). On the other hand, the temperature of -7°C has been determined as the temperature at which symptoms are observed in the leaves when the entire potted plantlet is subjected to frost. The observed 3°C difference between the experiment conducted on detached leaves and on potted plantlets may be attributed to the omission of certain variables and signals influenced by the root or stem when evaluating detached leaves (Karlova et al., 2021). Regarding the observation of the symptoms, it has been determined that clear manifestations are evident after 24 h, with no differences between the 24- and 172-h intervals. This timeframe coincides with the period during which cells affected by freezing lose their turgor, degrade, and lose their green color. Conversely, the unaffected cells remain turgid and maintain their natural green color (Hörtensteiner and Kräutler, 2011; Neri et al., 2020).

In the experiment with potted plantlets, no significant differences were observed among the studied cultivars. This can be attributed to the low number of evaluated plantlets per cultivar. Our results confirmed that a temperature of -7°C is optimal for evaluating frost tolerance when using plantlets. Despite both experiments (detached leaves and potted plantlets) yielding observable symptoms, the detached leaf experiment allowed us to work with a greater number of cultivars and achieve more repetitions in a shorter timeframe. Hence, to evaluate cultivars, the detached leaf methodology emerges as the most appropriate approach.

In our study, as in the previous ones, it is clearly observed how acclimated plants are less affected by frost damage compared to non-acclimated ones (Hashempour et al., 2014), although in field conditions and in the periods in which the frost events occur, it is difficult to determine the physiological stage of acclimation of different olive cultivars, so that the comparison is usually not reliable. This requires verifying frost tolerance under controlled conditions using homogeneously grown plant material in order to validate the results obtained in the field.

In this sense, for both acclimated and non-acclimated plants, ‘Frantoio’ was the most affected cultivar, confirming what was reported in previous studies (Bartolozzi and Fontanazza, 1999; Valverde et al., 2020). Besides that, since frost events can occur both in winter, when olive tree is fully acclimated, and in autumn or spring, when the tree can be not yet fully acclimated or de-acclimated, the frost tolerance evaluation must be done with both acclimated and non-acclimated plants. This response to frost could differ in the same cultivar depending on its acclimation (or de-acclimation) status. Because of this, the same cultivar could be classified as more or less tolerant to frost depending on the level of acclimation and according to the moment and way of occurrence of the frost event.

An essential consideration in refining the methodology for assessing frost tolerance was ensuring uniform exposure within the freezer. That was crucial due to the subtle temperature gradient inside the freezer cabinet, which is dependent on the height at which the plant material was positioned, directly impacting the observed damages. Similar temperature gradients are also observed during cold episodes in the field, a phenomenon known as thermal inversion (Serman et al., 2014). Therefore, to ensure a consistent and homogeneous response, we determined that evaluations must be conducted at the same height, both in controlled environments and in field conditions.

The methodology for frost tolerance assessment based on the evaluation of symptoms over detached olive leaves subjected to freezing temperatures can be appropriate to classify olive tree cultivars according to their frost tolerance. Either fully expanded (mature) leaves or newly formed (young) ones are suitable for the evaluations, although mature leaves are recommended to ensure homogeneity. The identified optimal freezing temperature was -10°C, and the assessment of symptoms under high humidity conditions was best conducted within 24–48 h. Therefore, the temperature of -10°C ± 1°C resulted to be the critical one to detect a different frost susceptibility of the tested olive cultivar using detached leaves as bioassay.

The method, developed and refined, offers the potential to evaluate very easily and quickly the frost tolerance for several olive cultivars. It can also be adapted to wild olive trees and other species within the *Olea* genus. However, it is imperative to note that the effectiveness of the results hinges on the homogeneity of the plant material used for the assessment. Without uniformity, the findings may not accurately reflect the conditions observed in cultivars within commercial field settings.

Upon reviewing existing literature and recognizing the multifaceted nature of the factors involved, it becomes apparent that many previous studies overlook some of these elements. This oversight could contribute to certain discrepancies or inconsistencies in the reported results. Notably, factors such as the state of acclimation of the plant at the time, the way of occurrence of frost, and the specific cultivar wield substantial influence within this intricate equation (Hashempour et al., 2014; Guerra et al., 2015).

## Data availability statement

The original contributions presented in the study are included in the article/supplementary material. Further inquiries can be directed to the corresponding authors.

## Author contributions

PV: Formal analysis, Investigation, Methodology, Writing – original draft, Writing – review & editing. EL: Conceptualization, Methodology, Supervision, Writing – review & editing. VG: Methodology, Supervision, Writing – review & editing. MG-L: Formal analysis, Investigation, Writing – review & editing. DN: Conceptualization, Funding acquisition, Supervision, Writing – review & editing.
